# Supplementary material for: Inadequacy of fluvial energetics for describing gravity current autosuspension
Source: Nat Commun. 2023 Apr 21;14:2288. doi: 10.1038/s41467-023-37724-1 (PMC10121697; doi:10.1038/s41467-023-37724-1)
Supplement: Supplementary file 2 — Supplementary Information [file 41467_2023_37724_MOESM2_ESM.pdf]

Inadequacy of fluvial energetics for describing gravity current autosuspension

Sojiro Fukuda[1*], Marijke G. W. de Vet[1], Edward W. G. Skevington[1], Elena Bastianon[1], Roberto Fernández[1], Xuxu Wu[1], William D. McCaffrey[2], Hajime Naruse[3], Daniel R. Parsons[1], and Robert M. Dorrell[1]

[1]Energy and Environment Institute, University of Hull, Hull, United Kingdom
[2]School of Earth and Environment, University of Leeds, Leeds, United Kingdom
[3]Department of Geology and Mineralogy, Division of Earth and Planetary Sciences, Graduate School of Science, Kyoto University, Kyoto, Japan
[*]e-mail: S.Fukuda-2018@hull.ac.uk

Supplementary Note 1. Non-dimensional flow characterization

Here the quasi-equilibrium flow condition is assumed, where the mean flow parameters do not vary in time and space but the turbulent fluctuation from mean flow parameters are still present. To describe these flow states, coordinate systems, and flow characteristics are introduced in this section. $\mathbf{x} = (x, y, z)$ denotes the spatial coordinate of the given point, where x is the streamwise direction, y is the lateral direction, and z is the bed-normal direction ($z = 0$ at the bed). Flow velocity is denoted by $\mathbf{u}(\mathbf{x},t) = (u, v, w)$. Volumetric flow concentration at a given height is denoted by $\phi(z)$. Reynolds averages[1] are introduced here to describe the mean flow characteristics and their fluctuations as follows

$$u(\mathbf{x},t) = \langle u(\mathbf{x},t)\rangle + u'(\mathbf{x},t), \quad v(\mathbf{x},t) = \langle v(\mathbf{x},t)\rangle + v'(\mathbf{x},t), \tag{1}$$

$$w(\mathbf{x},t) = \langle w(\mathbf{x},t)\rangle + w'(\mathbf{x},t), \quad \phi(\mathbf{x},t) = \langle \phi(\mathbf{x},t)\rangle + \phi'(\mathbf{x},t). \tag{2}$$

where $\langle\cdot\rangle$ denotes the temporal Reynolds average and primes denote the Reynolds fluctuations ($\langle u'\rangle = \langle v'\rangle = \langle w'\rangle = \langle \phi'\rangle = 0$). Then, the mean-flow field of quasi-equilibrium turbidity current is defined by introducing depth-averaged parameters as follows

$$U = \frac{1}{h(x)}\int_0^\infty \langle u(\mathbf{x})\rangle \mathrm{d}z, \quad V = \frac{1}{h(x)}\int_0^\infty \langle v(\mathbf{x})\rangle \mathrm{d}z, \quad W = \frac{1}{h(x)}\int_0^\infty \langle w(\mathbf{x})\rangle \mathrm{d}z, \quad \Phi = \frac{1}{h(x)}\int_0^\infty \langle \phi(\mathbf{x})\rangle \mathrm{d}z, \tag{3}$$

where $h(x)$ denotes flow depth. It should be noted that it is assumed that flow parameters do not vary in time for quasi-equilibrium turbidity currents. Flow depth is defined as the height at which both flow velocity and concentration vanish, which is

$$h(x) = \max\left[z|_{\langle u\rangle=0}, z|_{\langle \phi\rangle=0}\right], \tag{4}$$

where $z|_{\langle u\rangle=0}$ is the flow depth above which flow velocity becomes negligible and $z|_{\langle \phi\rangle=0}$ is the flow depth above which concentration difference between the floa and the ambient fluid becomes negligible. In the ideal condition, $z|_{\langle u\rangle=0} = z|_{\langle \phi\rangle=0}$. However, in the actual measurement of experiments, $z|_{\langle u\rangle=0}$ is not always the same value as $z|_{\langle \phi\rangle=0}$ for various reasons such as i) the effects of the non-zero flow velocity of ambient fluid, ii) the error of velocity or density measurements at the upper layer of the flow, and iii) error due to the extrapolation methodology. In particular, for turbidity currents, the vertical gradient of concentration profiles tends to be very small and show gradual transition to the ambient fluid. Thus, measurement error near the upper boundary could cause a significant error of the estimation of $z|_{\langle \phi\rangle=0}$. The majority of flume experiments use a few to a dozen siphon tubes to measure the flow concentration. On the other hand, the majority of compiled experiments measured vertical velocity profiles by using UVP or ADV, which have a much higher resolution than a siphon array. However, some of the flume experiments also have low resolution of velocity profiles, such as Michon et al.[2], Altinakar et al.,[3] and Tesaker[4] (Table 1). Thus, the accuracy of $z|_{\langle u\rangle=0}$ and $z|_{\langle \phi\rangle=0}$ varies between each experiment. In this study, the zero-velocity and zero-concentration heights are carefully compared, and the more reliable one was employed as flow depth (see below for detailed procedures).

To describe the flow state, the following non-dimensional flow parameters are introduced. Firstly, the Richardson number, *Ri* the ratio of the buoyancy and the flow shear stress, is given as,

$$\mathrm{Ri} = \frac{-g(\partial\langle\rho\rangle/\partial z)}{\rho_\mathrm{a}(\partial\langle u\rangle/\partial z)^2} \simeq \frac{g\Delta\rho/h}{\rho_\mathrm{a}U^2/h^2} = \frac{gR\Phi h}{U^2} \tag{5}$$

where ρ is the flow density, ρ_a is the density of the ambient fluid, and $\Delta\rho$ is the density difference between the flow and the ambient fluid ($\langle\rho\rangle - \rho_a$). The shear velocity, u_*, is calculated using the logarithmic law local to the bed,

$$\frac{d\langle u\rangle}{dz} = \frac{u_*}{\kappa z} \tag{6}$$

The skin drag coefficient, C_D, is defined as

$$C_D = \frac{u_*^2}{U^2}. \tag{7}$$

In this study, the particle settling velocity, w_s, for relatively coarse material (> 100μm) is prescribed by an empirical formula covering a combined viscous plus bluff-body drag law for natural irregular sand grains[5],

$$w_s = \frac{\nu}{d_{50}}\left[(10.36^2 + 1.049D_s^3)^{\frac{1}{2}} - 10.36\right]. \tag{8}$$

where d_{50} is the median grain size, and $D_s = (g\Delta\rho/\rho\nu^2)^{1/3}d_{50}$ is the dimensionless particle diameter, ν is the fluid viscosity. For the finer particles (< 100μm), Stokes' law is applied,

$$w_s = \frac{Rgd_{50}^2}{C_1\nu} \tag{9}$$

where R is specific gravity and C_1 is a constant with a theoretical value of 18.

Supplementary Note 2. Data compilation

source	TYPE	Slope (%)	Material	d_{50} (μm)	Measurement tools
Michon et al.[2]	I	0.3–3.6	Kaolinite	14.6	$\langle u\rangle$: MPCM<br>$\langle\phi\rangle$: Siphon array (4–12)
Tesaker[4]	I	5.0–12.5	Quartz<br>Kaolinite	360–410<br>1.1–1.5*4	$\langle u\rangle$: Velocity meters*6(3)<br>$\langle\phi\rangle$: Siphon array (3)
Altinakar[3]	I	1.0–2.96	Quartz<br>Salt	14 / 32<br>–	$\langle u\rangle$: MPCM<br>$\langle\phi\rangle$: Siphon array (16)
Garcìa[6]	I	8	Silica	9	$\langle u\rangle$: MPCM<br>$\langle\phi\rangle$: Optical probes
Packman & Jerolmack[7]	I	1.0	Quartz<br>Kaolinite	12<br>1.3	$\langle u\rangle$: ADV<br>$\langle\phi\rangle$: Siphon array (6)
Amy et al.[8]	I	5.2	Glycerol	–	$\langle u\rangle$: UVP array (8)<br>$\langle\phi\rangle$: Siphon array (5)
Islam & Imran[9]	I & II	0.0-8.0	Silt / Salt	25	$\langle u\rangle$: ADV<br>$\langle\phi\rangle$: Siphon array (20)
Sequeiros et al.[10]	I	0.85–5.0	Salt*3	–	$\langle u\rangle$: ADV<br>$\langle\phi\rangle$: Siphon array (10)
Sequeiros et al.[11]	I	5.0	Salt*3	–	$\langle u\rangle$: ADV<br>$\langle\phi\rangle$: Siphon array (10)
Cartigny et al.[12]	II	12.3–21.3	Quartz	160	$\langle u\rangle$: Angled UVP<br>$\langle\phi\rangle$: –
Varjavand et al.[13]	I	1.25	Kaolinite	13.4	$\langle u\rangle$: Angled UVP<br>$\langle\phi\rangle$: Siphon array (14)
Fedele et al.[14]	I	8.7–17.6	Salt	–	$\langle u\rangle$: ADV<br>$\langle\phi\rangle$: Siphon array (20)
Breard & Lube[15]	I (PDCs)	15.8	Ignimbrite	250	$\langle u\rangle$: PIV<br>$\langle\phi\rangle$: LC & PT

Leeuw et al.[16]	I & II	15.8–19.4	Sand	141	$\langle u \rangle$: Angled UVP<br>$\langle \phi \rangle$: Siphon array (4)
Leeuw et al.[17]	II	19.4	Sand	131	$\langle u \rangle$: Angled UVP<br>$\langle \phi \rangle$: –
Hermidas et al.[18]	II	10.5–16.7	Quartz<br>Kaolinite	150/46<br>0.18	$\langle u \rangle$: Angled UVP<br>$\langle \phi \rangle$: –
Sequeiros et al.[19]	I	9.0	Plastic	57	$\langle u \rangle$: Angled UVP<br>$\langle \phi \rangle$: Siphon array (11)
Eggenhuisen et al.[20]	I	7.0–14.0	Sand	130	$\langle u \rangle$: Angled UVP<br>$\langle \phi \rangle$: Siphon array (4)
Farizan et al.[21]	I	1.0	Kaolinite	11	$\langle u \rangle$: ADV<br>$\langle \phi \rangle$: ADV
Kelly et al.[22]	II*1	3.5	Salt	–<br>–	$\langle u \rangle$: ADV<br>$\langle \phi \rangle$: –
Koller et al.[23]	I*2	0.9–2.6	Salt	–	$\langle u \rangle$: UVP array (10)<br>$\langle \phi \rangle$: Siphon array (6)
Brosch & Lube[24]	I (PDCs)	10.5	Ignimbrite	245	$\langle u \rangle$: PIV<br>$\langle \phi \rangle$: LC & PT
Pohl et al.[25]	I	14.1	Quartz	133	$\langle u \rangle$: Angled UVP<br>$\langle \phi \rangle$: Siphon array (4)
Simmons et al.[26]	I	0.7	Silt	9.9–11*5	$\langle u \rangle$: ADCPs<br>$\langle \phi \rangle$: ADCPs

MPCM: Micro-Propeller Current-Meter; UVP: Ultrasonic Velocity Profiler;
ADV: Acoustic Doppler Profiler; ADCPs: Acoustic Doppler Current Profilers
PIV: Particle Image Velocimetry; LC: s-beam Load Cell: PT: Pressure Transducer

Supplementary Table 1. Summary of compiled sources. The figures in the parentheses represent the number of measurement devices in the array. *1 Numerical simulation of Reynolds-averaged Navier-Stokes model. *2 Limited information about concentration profiles reported in this study. *3 There are also experiments with sediment, but the availability of flow profiles is limited. *4 Original reported values of particle size from hydrometer analysis. *5 d_{50} from Event 1, 4, and 5 from the original source. *6 Velocity meters are special equipment that are designed for their particular study.

The compiled published sources are listed in Table 1. In total, 22 laboratory experiments, 1 numerical simulation, and 1 direct-observation source were gathered. Since each source uses different tools for vertical-profile measurement, the data are compiled carefully. For example, the velocity measurement of flume experiments can be categorized into 5 different types: micro-propeller current-meters, velocity meters, UVPs (Ultrasonic Velocity Profiler), ADVs (Acoustic Doppler Velocity probes). For both UVPs and ADVs, each source used a different number of probes, sampling rate, and angle of mounting. The vertical flow profiles reported in each source are based on interpolation and extrapolation, the methods differing between the sources. To perform consistent analysis, here, only the original measurement points are extracted from each sources to minimize the errors. Then, a consistent interpolation and extrapolation method are applied to recover the full vertical profiles. The experiments in Varjavand et al[13] and Farizan et al.[21] are designed to analyze the effects of an obstacle to the flows. The experiments with obstacles are excluded from the analysis and only the experiments without obstacles are used.

Particle-size distribution analyses The potential error of particle size due to the different particle size analyses are carefully considered. Settling velocity, w_s, plays an important role in the required work done to keep sediment in suspension, $B = gR\Phi h w_s$. The particle size analysis methods in the compiled sources are either sieving combined with hydrometer method (SHM) or the laser diffraction method (LDM). SHM measures the settling velocity of the particles in a liquid. In SHM, the particles are assumed to be spherical and their sizes are calculated based on Stokes' law[27]. On the other hand, in LDM, the measured particles size becomes equivalent to that of a sphere giving the same diffraction as the particles. LDM can process each sample faster and provide more detailed information such as the number of particles and surface area, compared with SHM[28]. Further, LDM provides high repeatability and reproducibility[29].

Extensive comparison of SHM and LDM[28,30–36] has shown a large discrepancy between the measured proportion of clay-size particles. Although the discrepancy becomes negligible for sand-size particles in most cases, when comparing the size of the largest particles in sample of silt to clay-size particles, the error can be up to two orders of magnitude[33]. The

particle-size error of two orders of magnitude can result in up to 4 order of magnitude errors of settling velocity (Eq.(9)). Those measurement discrepancies are attributed to the density difference and shape variation of particles[37].

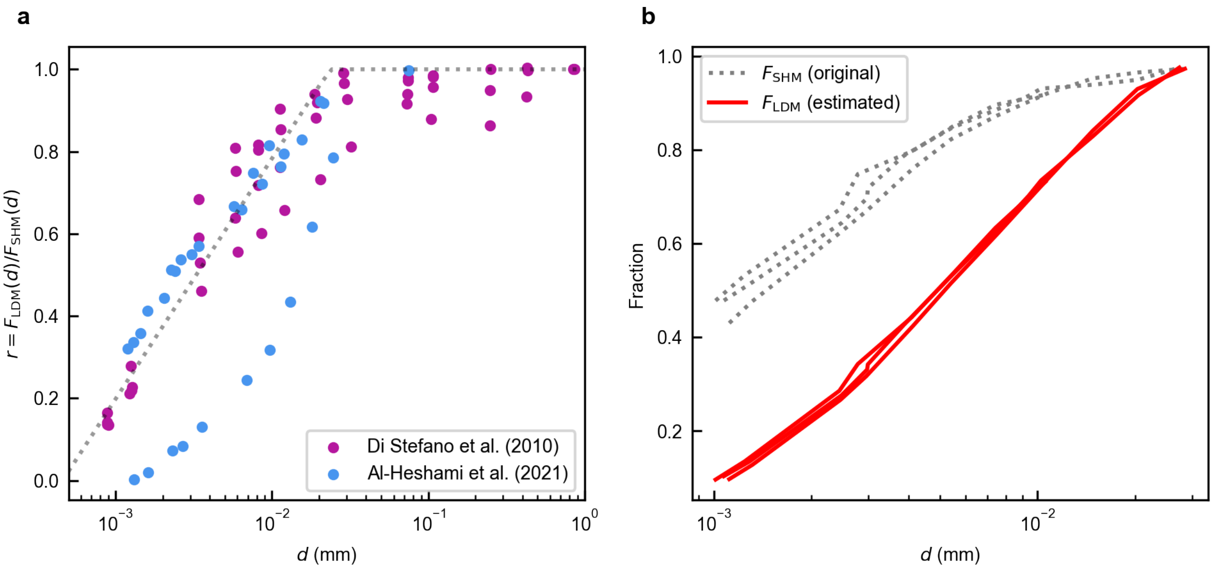

Supplementary Figure 1. a) The ratio of particle sizes as measured by the Laser Diffraction method to that of the hydrometer analysis method. Gray dotted line represents the fitted curve. b) Modified particle-size distribution of Tesaker[4].

In this study, particle size distribution from LDS is regarded as more reliable data than the SHM. Tesaker's experiment[4] used SHM to measure the particle size distribution of their clay material; the reported median particle size varies between 0.8–2.3 which is likely to be a significant underestimate. To mitigate this systematic error due to the difference of methodology, in this study, the following modification of reported particle size is conducted.

Firstly, cumulative particle distribution curves of similar materials to that used by Tesaker (Kaolinite clay) are extracted from the particle-size comparison studies between SHM and LDM[34,35]. Since the particle-size discrepancy between SHM and LDM could show different trends based on the type of clay minerals, only the datasets in which material is mainly composed of Kaolinite were chosen. The cumulative particle distribution curve, $F(d_i)$ is defined as in

$$F(d_i) = \sum_{k=0}^{i} f(d_k) \tag{10}$$

where d_i is the characteristic particle size of the ith bin, N is the totall number of bins, and $f(d_i)$ denotes the fraction of the ith particle size class which is given as

$$f(d_i) = \frac{q(d_i)}{\sum_k q(d_k)} \tag{11}$$

where $q(d_i)$ denotes the measured weight or volume of the particles in the ith size bin. Let $r(d)$ denote the ratio of the cumulative fraction of particles measured by LDM to the one measured by SHM, such that

$$F_{\text{LDM}}(d) = r(d) F_{\text{SHM}}(d). \tag{12}$$

Supplementary Figure 1a shows the calculated best fit function $r(d)$ from the compiled sources[34,35]; $r(d)$ increases monotonically as d increases until r reaches to unity at $d \simeq 3 \times 10^{-2}$.

Here, the relationship between r and $\log d$ for fine particles ($d < 3 \times 10^{-2}$) is approximated by the linear curve fitting with the least-square method (Gray dotted line in Supplementary Fig. 1a). Then, assuming $r \leq 1$, the empirical formula of r is given as

$$r(d) = \min\left[a \log_{10} d + b, 1\right] \tag{13}$$

where the correlation coefficients are $a = 0.58 \pm 0.04$ and $b = 1.95 \pm 0.09$. Then, F_{LDM} of Tesaker's experiments[4] is estimated from the measured F_{SHM} by Equation (12) and (13) (Supplementary Fig. 1b). Consequently, d_{50} shifted from around 1×10^{-3} mm to 4.98×10^{-3} (Supplementary Fig. 1b). In the analysis of the main text, 4.98×10^{-3} is used as the median particle size of Tesaker's experiments.

Extraction of original measurement points Depending on the availability of the dataset, different procedures were applied to extract data from the source. There were two cases: i) original raw measurement data (such as UVP measurements data) is available, ii) only figures of measured data points and interpolated and extrapolated profiles are available. In case (i), the raw data are directly used to reconstruct the flow profiles. In case (ii), the original measurements of vertical velocity and concentration are extracted by using a graph-read software (https://graphclick.en.softonic.com/mac). However, some of the sources have less than three points of velocity or concentration, which is not enough to apply the extrapolation method. Here, assuming that the potential errors due to the choice of interpolation and extrapolation near the maximum velocity height are relatively small, a few data points are manually added near the original data points along the interpolated curve in the original source.

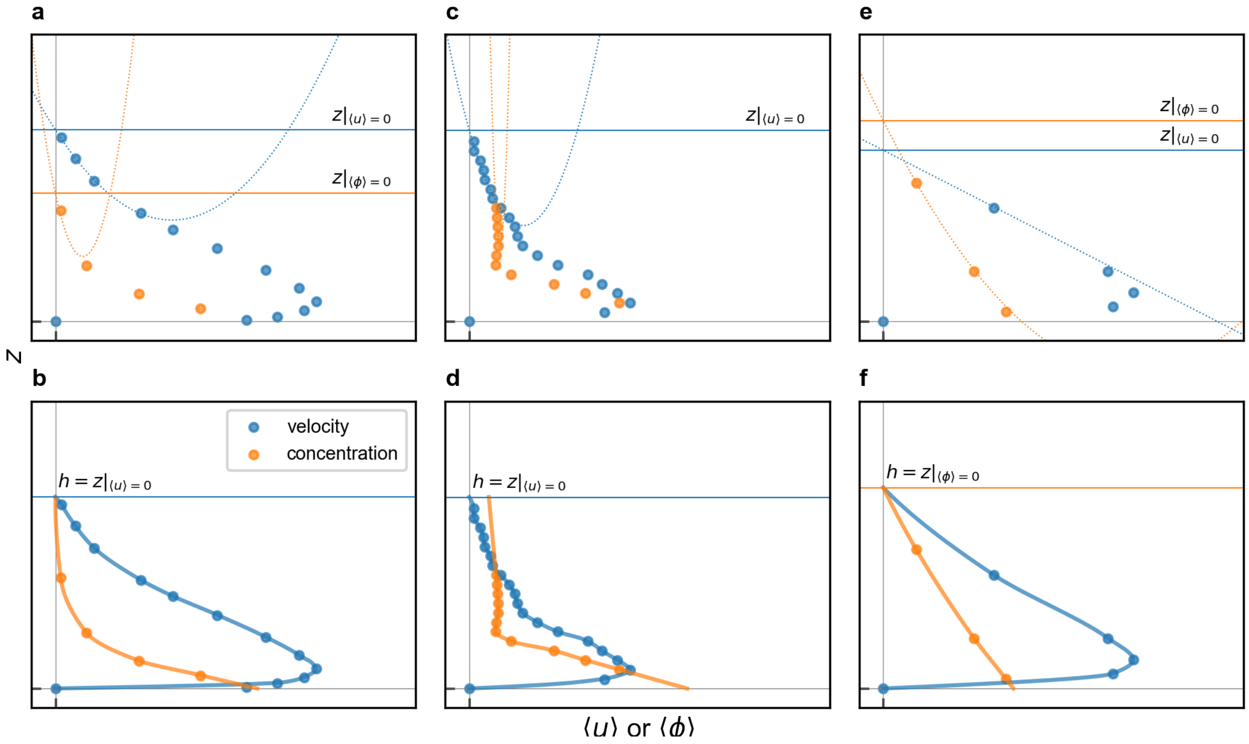

Supplementary Figure 2. Schematic diagram of interpolation and extrapolation procedures for three different cases. Blue and orange markers represent the original measurement values. a) b) $z|_{\langle \phi \rangle = 0} < z_{u,n}$. c) d) $z|_{\langle u \rangle = 0} < z|_{\langle \phi \rangle = 0}$. e) f) $z_{u,n} < z|_{\langle \phi \rangle = 0} < z|_{\langle u \rangle = 0}$. a) c) e) Schematics of quadratic curve fitting for flow height estimation. Fitted curves are represented by dotted curves. b) d) f) Schematics of resultant flow profiles.

Interpolation and extrapolation of flow profile The overall procedure can be summarized into three steps: i) data formatting, ii) estimation of flow height and near-bed concentration, and iii) interpolation and extrapolation. Details of each step are described in this section.

The first step is data formatting. Let $P_{\langle u \rangle} = \left[\mathbf{x}_{\langle u \rangle,1}, \mathbf{x}_{\langle u \rangle,2}, \ldots \mathbf{x}_{\langle u \rangle,n}\right]$ and $P_{\langle \phi \rangle} = \left[\mathbf{x}_{\langle \phi \rangle,1}, \mathbf{x}_{\langle \phi \rangle,2}, \ldots \mathbf{x}_{\langle \phi \rangle,m}\right]$ denote the extracted values of velocity and concentration. n and m denote the number of extracted measurement points of velocity and concentration respectively. $\mathbf{x}_{\langle u \rangle,i} = (\langle u \rangle|_{z=z_{u,i}}, z_{u,i})$ and $\mathbf{x}_{\langle \phi \rangle,i} = (\langle \phi \rangle|_{z=z_{\phi,i}}, z_{\phi,i})$ denote the ith coordinate of extracted points. The data points are ordered from lowest to highest so that $z_i < z_{i+1}$. Thus, $z_{u,1}$ and $z_{\phi,1}$ are the lowest original measurement heights and $z_{u,n}$ and $z_{\phi,m}$ are the highest measurement heights. As boundary conditions, the following relationship is assumed for velocity profiles in this study.

$$\langle u \rangle|_{z=0} = \langle u \rangle|_{z=h} = 0, \tag{14}$$

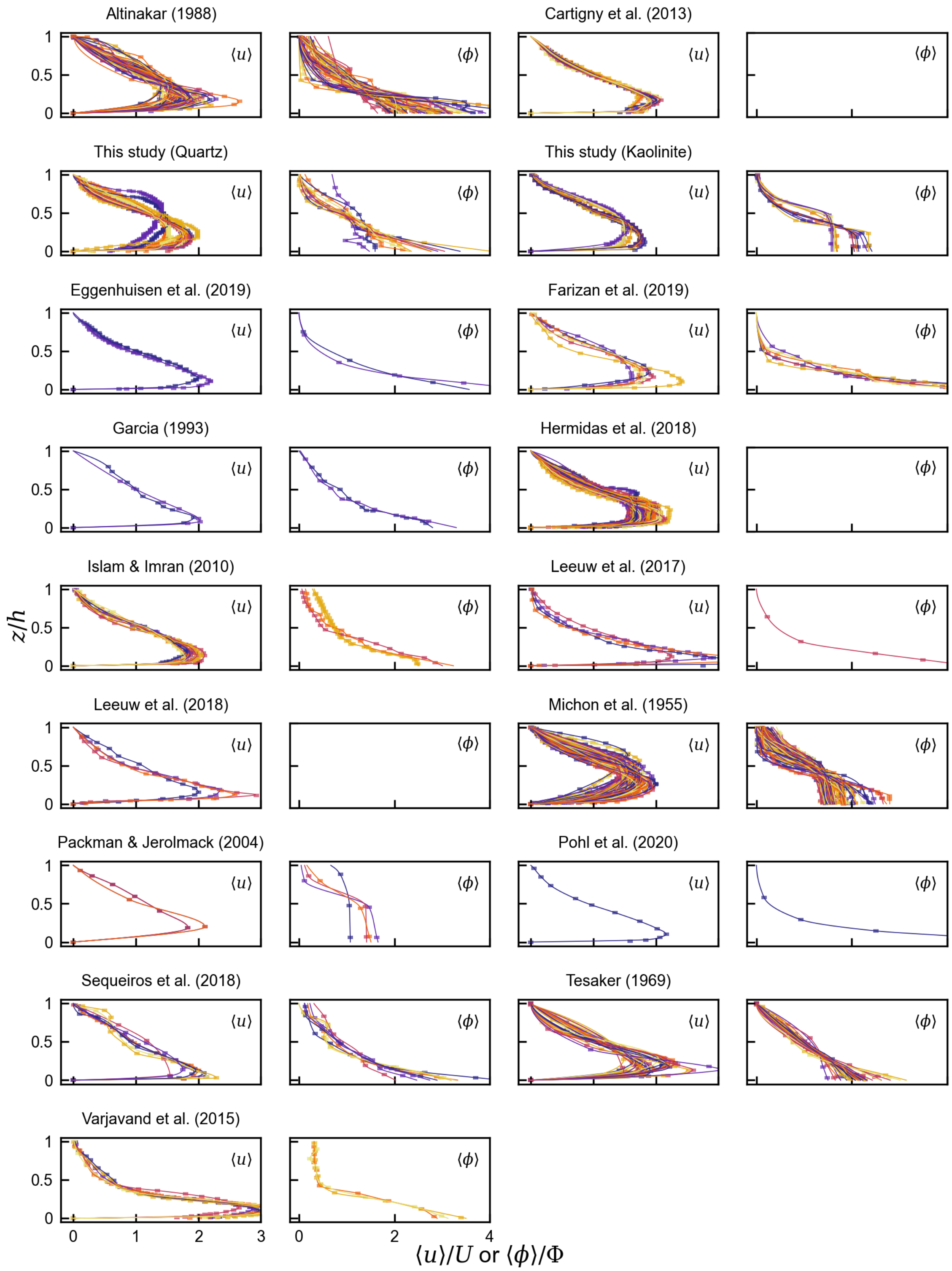

Supplementary Figure 3. Interpolated and extrapolated flow profiles of the non-conservative flows from each source. For each source, velocity profiles are plotted on the left, and concentration profiles are on the right. X-axes are either flow velocity or flow concentration which is normalized by depth-averaged values, and Y-axes are the distance from the bed normalized by flow height.

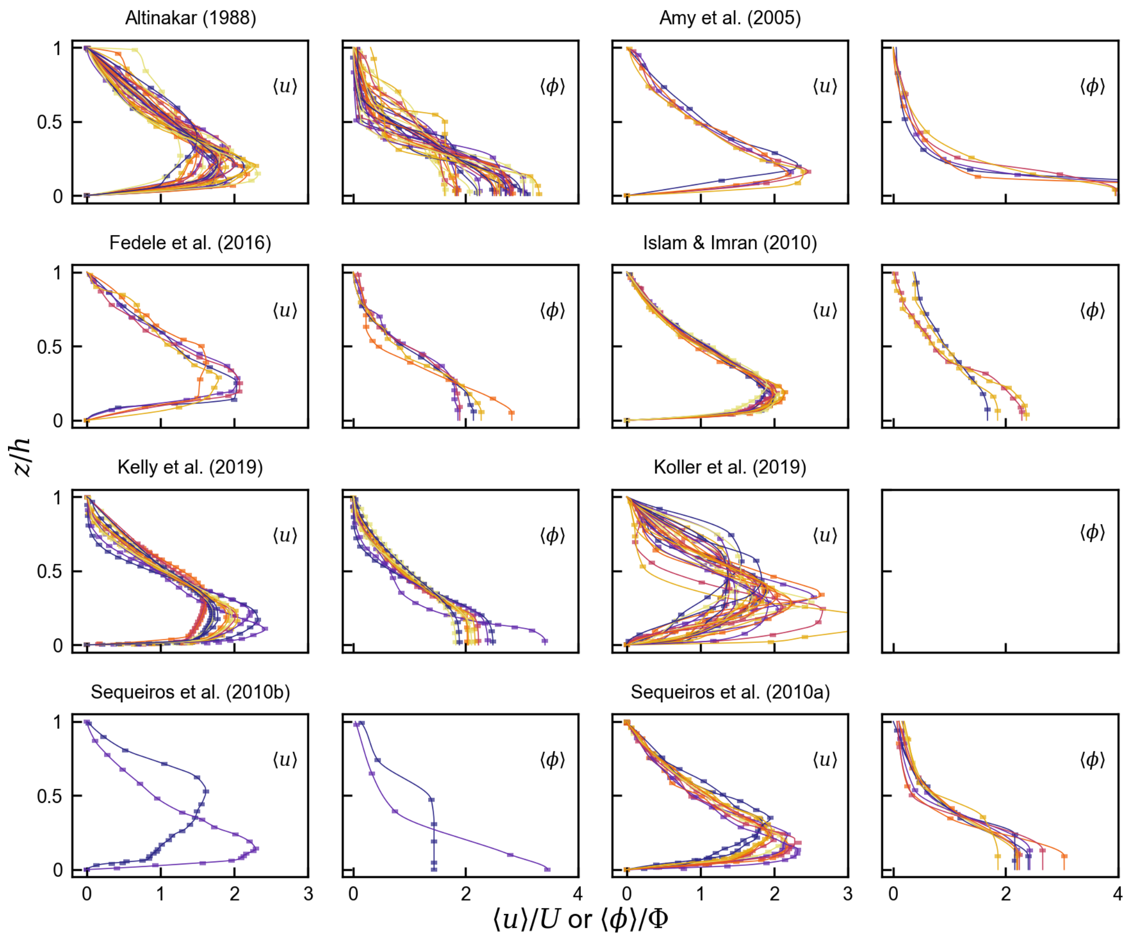

Supplementary Figure 4. Interpolated and extrapolated flow profiles of the conservative flows from each source. For each source, velocity profiles are plotted on the left, and concentration profiles are on the right figure. X-axes are either flow velocity or flow concentration which is normalized by depth-averaged values, and Y-axes are the distance from the bed normalized by flow height.

To impose the lower boundary condition, an artificial data point $\mathbf{x}_{\langle u\rangle,0} = (0,0)$ is inserted into $P_{\langle u\rangle}$.

The next step is to estimate the flow height. The estimation of the flow height is performed in two steps: i) calculation of $z|_{\langle u\rangle=0}$ and $z|_{\langle\phi\rangle=0}$, and then ii) evaluation of flow height based on the comparison between $z|_{\langle u\rangle=0}$ and $z|_{\langle\phi\rangle=0}$. Firstly, interpolation of $P_{\langle u\rangle}$ and $P_{\langle\phi\rangle}$ is conducted using the PchipInterpolator function from Scipy, a Python package. This function provides a piecewise cubic Hermite interpolation[38]. One of the advantages of this interpolation method is that it does not overshoot even when the data is not smooth. Further, in this interpolation method, the original data points are always on the interpolated curve, which means that potential oversimplification is minimal. Let $f_{\langle u\rangle}(z)$ and $f_{\langle\phi\rangle}(z)$ denote the interpolated functions. Then, to obtain enough data points for processing, 500 vertically uniform data points are extracted from the interpolated function,

$$P_{f,\langle u\rangle} = \left[(f_{\langle u\rangle}(z_{fu,0}), z_{fu,0})...(f_{\langle u\rangle}(z_{fu,499}), z_{fu,499})\right], \quad P_{f,\langle\phi\rangle} = \left[(f_{\langle\phi\rangle}(z_{f\phi,0}), z_{f\phi,0})...(f_{\langle\phi\rangle}(z_{f\phi,499}), z_{f\phi,499})\right]. \tag{15}$$

It should be noted that the points $(f_{\langle u\rangle}(z_{fu,499}), z_{fu,499})$ and $(f_{\langle\phi\rangle}(z_{f\phi,499}), z_{f\phi,499})$ correspond to the highest original data points, $\mathbf{x}_{\langle u\rangle,n}$ and $\mathbf{x}_{\langle\phi\rangle,m}$ respectively. Quadratic-function curve fitting is conducted against a upper part of the velocity profile to obtain $z|_{\langle u\rangle=0}$ and $z|_{\langle\phi\rangle=0}$ (Supplementary Fig. 2a,c,e). For the velocity profile, the range of curve fitting is between $z_{fu,u_{\max}}$ and $z_{fu,499}$, where $z_{fu,u_{\max}}$ is the nearest data point to the velocity maximum. For the concentration profile, the range is between $z_{f\phi,249}$ and $z_{f\phi,499}$, where $z_{f\phi,249}$ denotes the data point at the middle height of interpolated region. The quadratic functionas, g_u and g_ϕ are given as,

$$g_u(u) = a_1\langle u\rangle^2 + a_2\langle u\rangle + a_3, \quad g_\phi(\phi) = b_1\langle\phi\rangle^2 + b_2\langle\phi\rangle + b_3. \tag{16}$$

where (a_1, a_2, a_3) and (b_1, b_2, b_3) are the coefficients of best-fit curves estimated by the least-square method. The conditions, $a_1 > 0$ and $b_1 > 0$ are imposed to avoid unrealistic profile shapes. Then, $z|_{\langle u\rangle=0}$ and $z|_{\langle\phi\rangle=0}$ are obtained as,

$$z|_{\langle u\rangle=0} = g_u(0), \quad z|_{\langle\phi\rangle=0} = g_\phi(\phi_{\text{ambient}}). \tag{17}$$

where ϕ_{ambient} is the concentration of the ambient fluid. Then, the flow height is given by,

$$h = \begin{cases} z|_{\langle u\rangle=0} & \text{when } z|_{\langle u\rangle=0} \le z|_{\langle\phi\rangle=0} \text{ or } z|_{\langle\phi\rangle=0} < z_{u,n}, \\ z|_{\langle\phi\rangle=0} & \text{when } z_{u,n} < z|_{\langle\phi\rangle=0} \le z|_{\langle u\rangle=0}. \end{cases} \tag{18}$$

$z|_{\langle\phi\rangle=0} < z_{u,n}$ sometimes happens when the number of data points in the density profile are inadequate. In this study, the original velocity measurements are regarded as more reliable than the extrapolated concentration curve. Thus, when $z|_{\langle\phi\rangle=0} < z_{u,n}$ (Supplementary Fig. 2a,b), the extrapolation of the concentration profile is discarded and $h = z|_{\langle u\rangle=0}$ is the flow depth. Then, a new velocity point at the top of the flow $\mathbf{x}_{\langle u\rangle,h} = (0,h)$ is inserted into P_u. For the concentration profile, the value of the concentration at the flow height, $\mathbf{x}_{\langle\phi\rangle,h}$ is not necessarily always ϕ_{ambient}. When $z|_{\langle u\rangle=0} \le z|_{\langle\phi\rangle=0}$, the flow concentration at the flow height is estiamted by a piecewise cubic Hermite interpolation of original measurement points with $(0, z|_{\langle\phi\rangle=0})$. Let $f'_\phi(z)$ denote the interpolated function. Then, the flow concentration at the flow height is given as $f'_{\langle\phi\rangle}(z=h)$.

The final step is the interpolation and extrapolation of the rest of the profiles. Extrapolation is required to estimate the near-bed concentration, $\langle\phi\rangle_{\text{bed}}$. For the non-conservative currents, the first-order polynomial curve fit is conducted against the lowest two data points. For the conservative flow, the lowest original measurement point $\langle\phi\rangle|_{z=z_{\phi,0}}$ is used as the near-bed concentration. Then, combining the original measurements points, near-bed points, and flow height points, the following completed data lists were obtained,

$$P'_{\langle u\rangle} = \left[\mathbf{x}_{\langle u\rangle,\text{bed}}, \mathbf{x}_{\langle u\rangle,1}, ...\mathbf{x}_{\langle u\rangle,n}, \mathbf{x}_{\langle u\rangle,h}\right], \quad P'_{\langle\phi\rangle} = \left[\mathbf{x}_{\langle\phi\rangle,\text{bed}}, \mathbf{x}_{\langle\phi\rangle,1}, ...\mathbf{x}_{\langle\phi\rangle,m}, \mathbf{x}_{\langle\phi\rangle,h}\right]. \tag{19}$$

Finally, by conducting piecewise cubic Hermite interpolation against $P'_{\langle u\rangle}$ and $P'_{\langle\phi\rangle}$, the full profile $\langle u\rangle(z)$ and $\langle\phi\rangle(z)$ were obtained. The full profiles for each source obtained by the methodology described here are plotted in Supplementary Figure 3, 4.

Turbidity currents from Congo canyon The real-world dataset from Congo canyon is extracted from the publication by Simmmons et al.[26]. Here, flow velocity and concentration structures are measured by Acoustic Doppler Current Profilers (ADCPs). In this study, to investigate the flow dynamics of quasi-equilibrium natural-scale turbidity currents, relatively long-duration (more than 5 days) events are chosen (Event 01, 04, and 05). Some long-duration events in which velocity and concentration fields drastically changed during the flow event (such as Events 8, 9, and 10 from the original source) are excluded.

Firstly, the moving averages of the velocity and concentration time series are calculated from each event (Supplementary Fig. 5). Then, the depth-averaged flow parameters, (flow velocity and concentration), are calculated from each averaged

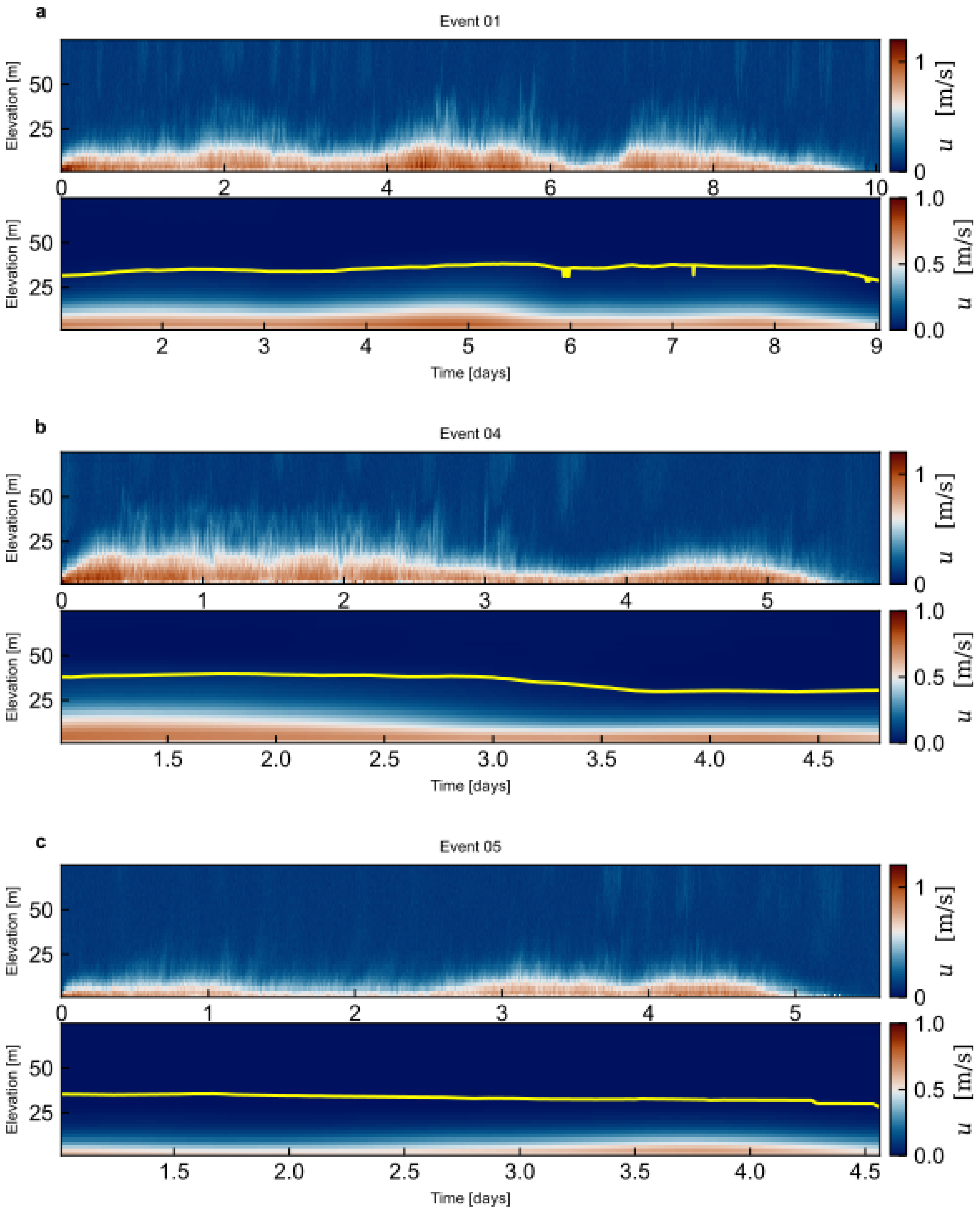

Supplementary Figure 5. Time series of original and moving-averaged velocity field of the turbidity currents in the Congo system: a) Event 01, b) Event 04, c) Event 05. Yellow lines are the estimated flow height.

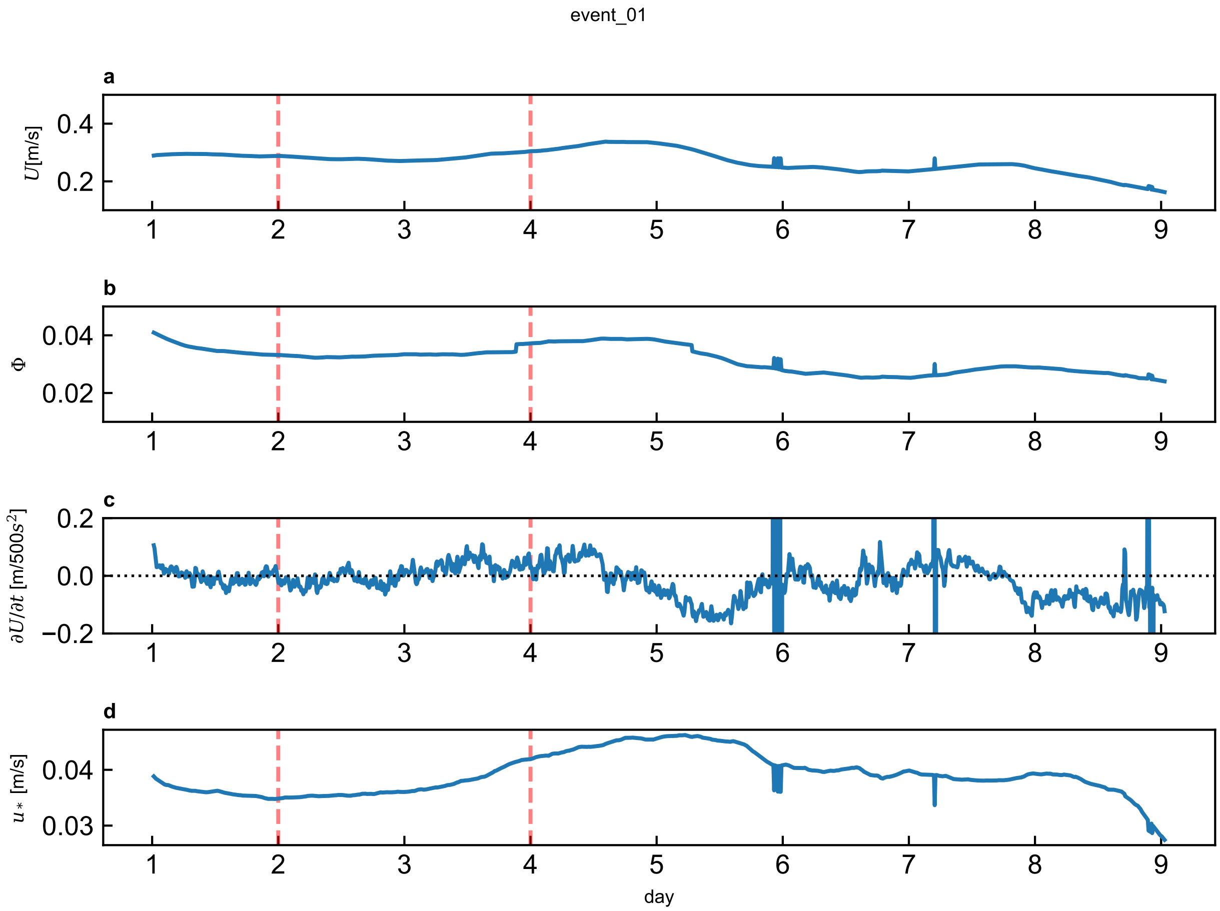

Supplementary Figure 6. Depth-averaged flow parameters of Event 01 in Congo canyon. a) Depth-averaged flow velocity, b) depth-averaged flow concentration, c) time gradient of depth-averaged flow velocity [m/500s^2] and, d) estimated shear velocity. The selected time interval is indicated by red dashed lines.

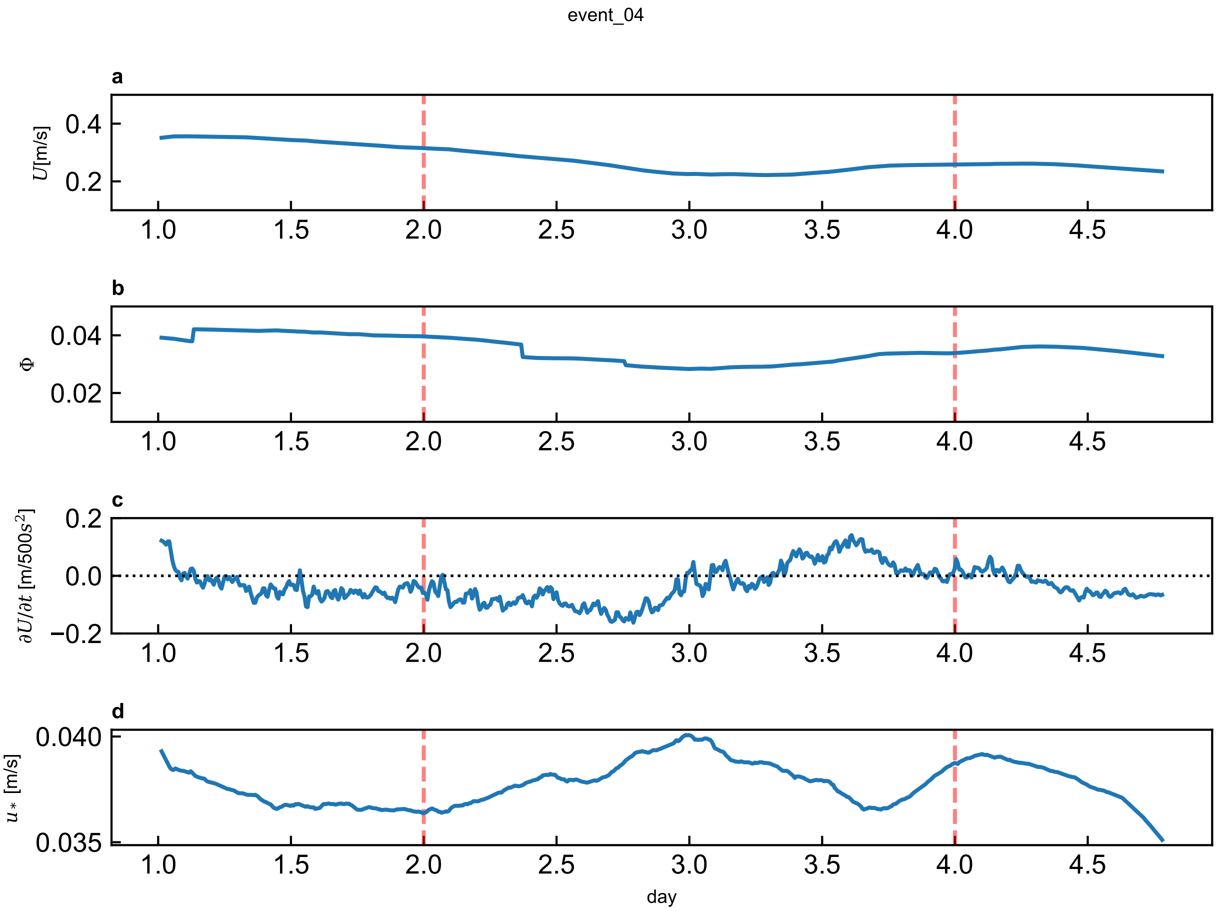

Supplementary Figure 7. Depth-averaged flow parameters of Event 04 in Congo canyon. a) Depth-averaged flow velocity, b) depth-averaged flow concentration, c) time gradient of depth-averaged flow velocity [m/500s^2] and, d) estimated shear velocity. The selected time interval is indicated by red dashed lines.

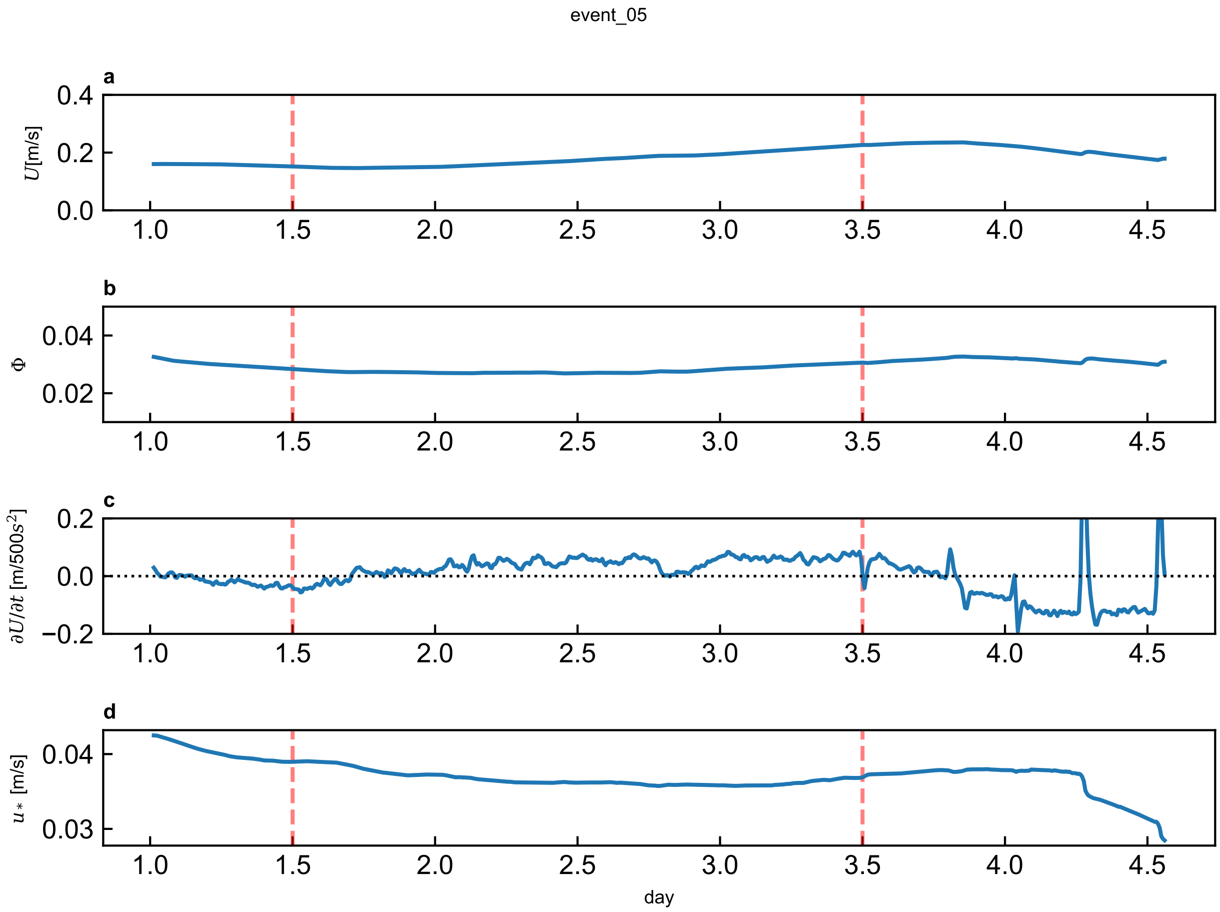

Supplementary Figure 8. Depth-averaged flow parameters of Event 05 in Congo canyon. a) Depth-averaged flow velocity, b) depth-averaged flow concentration, c) time gradient of depth-averaged flow velocity [m/500s^2] and, d) estimated shear velocity. The selected time interval is indicated by red dashed lines.

profile (Supplementary Fig. 6–8). Based on the gradient in time of the depth-averaged flow velocity, intervals of time for data compilation are selected so that the strongly accelerated or decelerated regions are excluded. Finally, from the selected window, mean depth-averaged flow parameters are calculated. To remove the background noise from each flow, the time-average background velocity with in the selected window is calculated for each run. Then velocity values lower than the background noise are excluded in the interpolation and extrapolation of profiles. The velocity relative to the time-averaged background velocity is extracted as the flow velocity measurements.

The original concentration profiles include some artifacts[26]. In events 1 and 4, sometimes concentration values suddenly increase near the interface with the ambient. Simmons et al.[26] assumed that this type of artefact is related to the backscatter from turbulent microstructure associated with gradients in either density, temperature, or salinity[39]. To exclude the artefacts, the peak value of the concentration near the upper flow interface is measured from Event 1 and 4. Then, in the upper half of the flow, the concentration lower than this peak is omitted; only the portion of the concentration data which is of higher concentration than the artefact is used for interpolation and extrapolation.

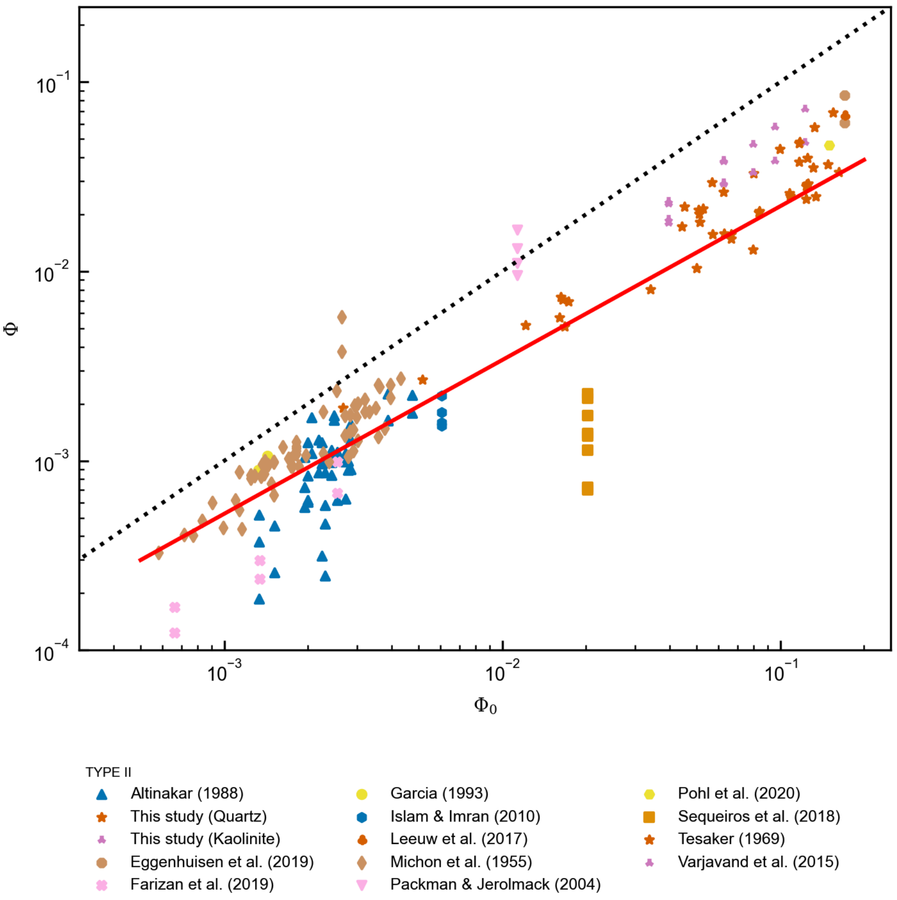

Supplementary Figure 9. Log-log plot of layer-averaged flow concentration and initial concentration of the sediment-water mixture in the mixing tank of each experiment. The regression line (gray dotted line) was fitted to logarithmic values of Φ_0 and Φ. The coefficient of determination is $R = 0.88$.

Estimation of depth-averaged concentration To estimate the depth-averaged sediment concentration, Φ, for the experiments in which the vertical concentration profiles are not available (TYPE III, see Table 1), the following empirical relationship between Φ and the initial sediment concentration in the mixing tank, Φ_0, are deduced, using orthogonal distance regression, from TYPE II data where both parameters are available (Supplementary Fig. 9). Then, Φ of TYPE III data is assumed to follow the obtained empirical formula,

$$\Phi \simeq 0.30 \times {\Phi_0}^{0.94} \tag{20}$$

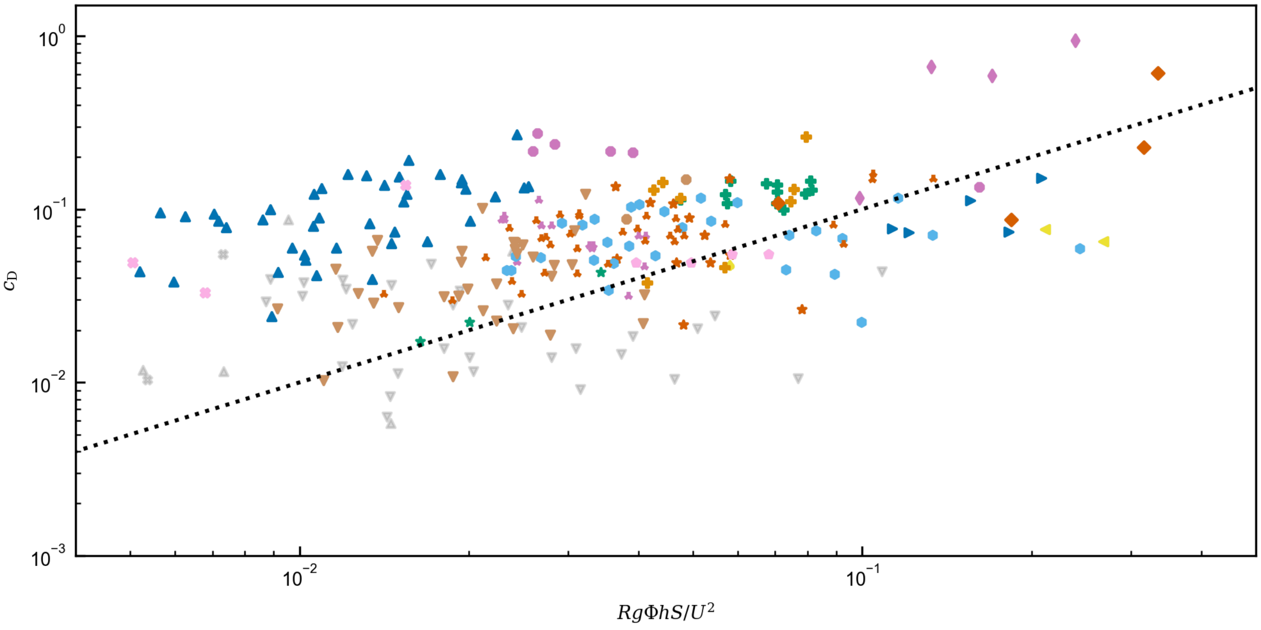

Supplementary Figure 10. Log-log plot of the estimated drag coefficient (Eq. 6–7) verses the top-hat drag coefficient[40] (Eq. 21). The black dotted line represents the ideal linear line ($C_\mathrm{D} = Rg\Phi hS/U^2$). The gray points indicate that those data points are excluded by the near-equilibrium criteria (Fig. 2 in the main text).

Drag coefficient In this study the drag coefficient, C_D is estimated from the velocity gradient (Eq. 6–7). On the other hand, when a flow is in equilibrium, top-hat (unstratified) shallow water models[40] give

$$C_\mathrm{D} = \frac{Rg\Phi hS}{U^2}, \tag{21}$$

where S denotes the energy slope. Here, we compare the drag coefficient values estimated from the velocity gradient (Eq. 7) and those values based on the top-hat equilibrium assumption (Eq. 21), see Supplementary Figure 10. The top-hat drag coefficient shows relatively better correlation with velocity-profile-based drag coefficient in the relatively large drag coefficient region ($Rg\Phi hS/U^2 \gtrsim 0.05$). While the flows with the low top-hat drag coefficient ($Rg\Phi hS/U^2 \lesssim 0.05$) show a relatively poor correlation with the velocity-profile-based drag coefficient. This serves to further stress the main conclusion, that the top-hat model does not accurately capture the dynamics of gravity currents.

Supplementary Note 3. Flume Experiments

The experiments are carried out in the flume laboratory in the Total Environmental Simulator at The DEEP. The flow properties (Table 1 in the main text) are chosen to fill the gaps between the data points from the compiled sources (Supplementary Fig. 11). A schematic and description of the experimental settings is given in the main text (Fig. 6 in the main text). Here, the preparation of the sediment-water mixture, and the bed profile measurement which is conducted to measure the aggradation rate, are elaborated.

Preparation of sediment-water mixture A large corn-shape mixing tank is built and used to generate turbidity currents (in total 1.36 m^3 including the region below the mixer which cannot be used; the total effective volume for the experimental run above the mixer is approximately 0.97 m^3). To keep the concentration vertically uniform, two different mixing systems are established: i) an electrical mixer with a set of 350 mm diameter impellers is inserted from the top of the tank which generates a strong vortex within the tank, and 2) a re-circulation piping system with a pump is connected from the bottom of the tank to the top of the tank which generates vertical circulation. Another piping system is connected with a slurry pump (Ebara DWO 300) from the fit on the sidewall of the tank to the flow diffuser inserted upstream of the perspex straight channel. The (pumped) input rate is tested by adjusting the in-line valve, reading the flow rate from an electromagnetic flowmeter during test runs. The sediment concentration in the mixing tank and in each flow are calculated from the wet and dry weight of collected samples. Finally, the dry sample was analyzed by a laser particle sizer (Malvern Mastersizer) to estimate the detailed particle size distribution.

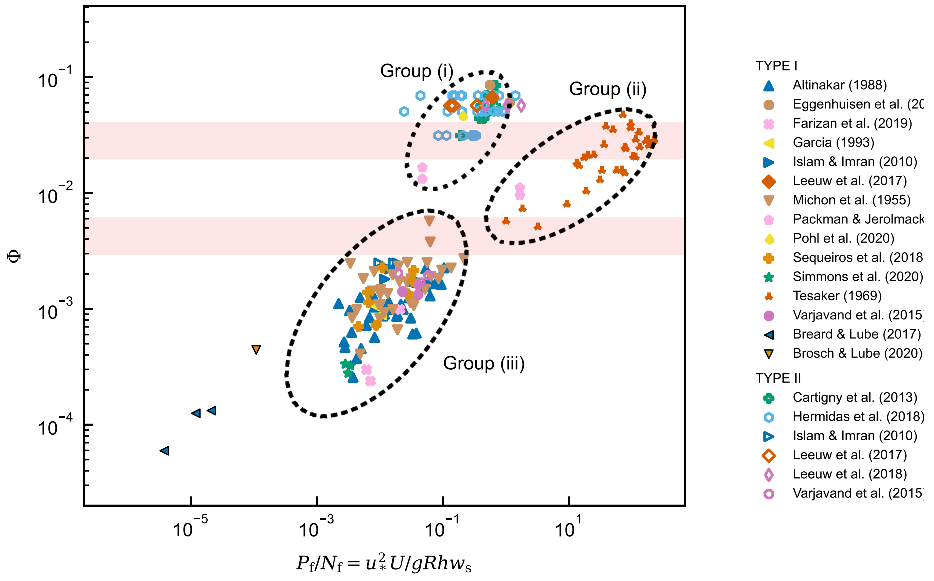

Supplementary Figure 11. Flow power plot with target flow concentration regions (red shaded area) of the flume experiments. Part of the experiments was aimed at filling the gap between groups of data points (i) and (ii). The rest of the experiments were aimed to supply the data between the group (ii) and (iii).

Bed profile Aggradation rate, for flow equilibrium, and bed depth is monitored by using the bed profiler from a single ADV (Acoustic Doppler Velocimeter) suspended above the flow. In addition to the ADV, 4 ultrasonic sensors (URSs) to monitor the aggradation rate are mounted. To reduce mobile bedload, and ADV measurement noise, experiments start with the unerodible bed of the Perspex channel. The maximum aggradation rate of the well-developed flow body observed in our experiments is 0.1 mm/s in run 11. It should be noted that for high concentrated runs (experiments 01–07 and 13–17, see Table 2 in the main text) both ADV and URSs failed to monitor the bed height during the flow events. For those runs, the aggradation rate is monitored from the videos that were captured by high-resolution GoPro cameras. As result, for those high concentration runs, deposition only occurred at the very end of the flow event (the tail of the flow) but we observed almost no deposition from the head and body of each flow where the velocity and density measurement are conducted.

Supplementary Note 4. Flow power plot

From the flow power theory[41,42] and the autosuspension criteria[43], the following proportionality is assumed

$$P_\mathrm{shear} \propto B_\mathrm{turb}, \qquad \text{where} \qquad P_\mathrm{shear} = -\int_0^\infty \langle u'w' \rangle \frac{\partial \langle u \rangle}{\partial z}\,\mathrm{d}z \qquad \text{and} \qquad B_\mathrm{turb} = \int_0^\infty \tilde{B}\,\mathrm{d}z = \int_0^\infty Rg\langle w'\phi' \rangle\,\mathrm{d}z, \tag{22}$$

where P denotes the shear production of the mean flow, and the buoyancy production term, B, denotes the work done by turbulence to keep sediment in suspension. For the fluvial flows, assuming $B_\mathrm{f} = \Phi N_\mathrm{f} = Rg\Phi h w_\mathrm{s}$ and the direct proportionality between total production, P_loss and the log-law production, $P_\mathrm{f} = {u_*}^2 U$,

$$\Phi \propto \frac{P_\mathrm{f}}{N_\mathrm{f}} = \frac{u_*^2 U}{Rghw_\mathrm{s}}, \tag{23}$$

is obtained.

Laboratory-scale turbidity currents data There are few data points in the dilute regime around $10^{-1} < P_\mathrm{f}/B_\mathrm{f} < 10^0$. This is not because the data is excluded due to the introduced equilibrium criteria (Fig. 2 in the main text) but simply the long-duration experiments of turbidity currents with flow concentration, $\Phi \sim 0.5\%$ are limited (Supplementary Fig. 12). Our flume experiments partially fill this data gap by adding three runs within the target concentration range.

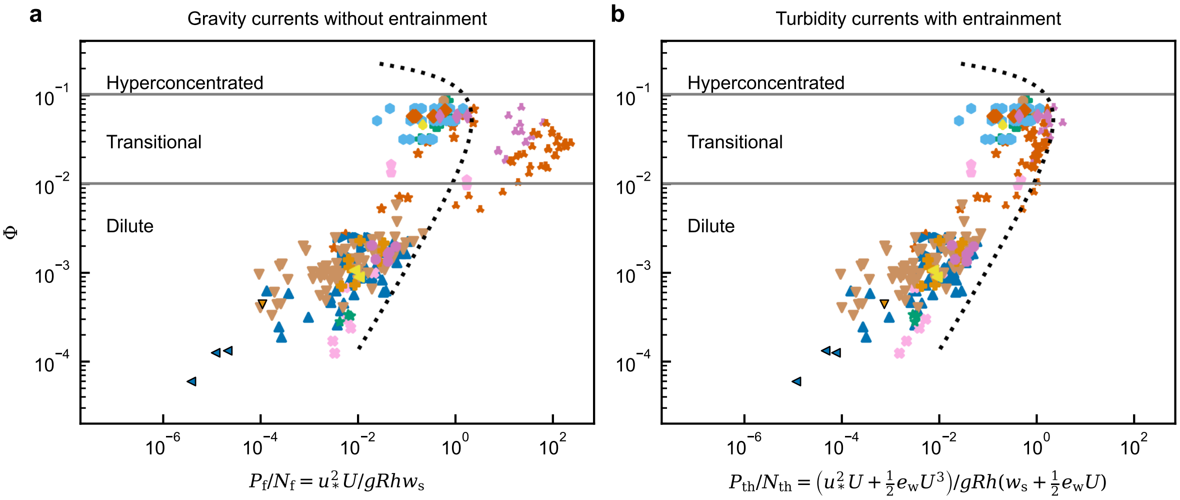

Supplementary Figure 12. Sediment transport capacity for gravity currents without data reduction. Sediment concentration, Φ, versus dimensionless flow power, P_f/N_f for: a) all gravity current data with the log-law production term, P_f; b) all gravity current data with the top-hat production term, P_{th}. Throughout, dilute, transitional and hyperconcentrated regimes are separated by gray solid lines. Parametric correlations of concentration and dimensionless flow power are depicted by black dotted curves. Symbol shapes as per Figure 1 in the main text.

Pyroclastic density currents The recent laboratory experiments[15,24] of pyroclastic density currents (PDCs) are gathered (see Table. 1). The same methodology of data compilation for turbidity currents is applied for PDCs. For the entrainment rate of air of pyroclastic density currents, the empirical equation[44]

$$e_w = 0.21 \mathrm{Ri_o}^{-1.1} \tag{24}$$

is used, where $\mathrm{Ri_o} = g' h \cos\theta / U^2$ is the overall Richardson number, g' is the reduced gravity, and θ denotes the angle of slope.

Fluvial flows The fluvial data from natural-scale rivers[45,46] and laboratory-scale experiments[47–56] are gathered (Fig. 3 in the main text and Supplementary Fig. 12a). The Yellow river data[46] are gathered from the figure from[57], using a graph reading software. Since the shear velocity values and raw velocity profiles of Yellow River data points are not available in the original source, the average skin drag coefficient, C_D of Yellow River data is estimated from the recent velocity measurement data[58]. The reported flow parameters from each source such as flow velocity, volumetric flow concentration, and median particle size are directly used for the calculation. For the detailed data compilation methodology for the rest of the data, see Dorrell et al.[59].

source	site
Vanoni[54]	Laboratory exp.
Brooks[56]	Laboratory exp.
Einstein & Chien[51]	Laboratory exp.
Vanoni & Nomicos[55]	Laboratory exp.
Nordin & Dempster[45]	Rio Grande
Guy et al.[47]	Laboratory exp.
Coleman[52]	Laboratory exp.
Lyn[53]	Laboratory exp.
Wan & Wang[46]	Yellow River
Ashida & Okabe[48]	Laboratory exp.
Cellino & Graf[49]	Laboratory exp.
Graf & Cellino[50]	Laboratory exp.

Supplementary Table 2. Summary of the compiled fluvial source.

Supplementary Note 5. Curve Fitting

In the main text, two types of regression analyses are conducted. For the simple linear regression, Orthogonal Distance Regression (ODR) method[60] is used. While Ordinary Least Squares (OLS) treat the data for explanatory variables (x-axis) as without error, minimising the distance from the response variable (y-axis) to the fitted curve, ODR accounts equally for errors of both explanatory and response variables, minimising the orthogonal distance between each data point and the fitted curve. All explanatory variables in the linear regressions in this study are expected to exhibit error due to (for example) the limitation of measurement tools such as UVP or siphon arrays. Thus, ODR is more suitable than OLS. In Table 1 in the main text, the standard error of each estimated parameter is given, which is calculated from the square root of the corresponding diagonal term in the parameter covariance matrix. To infer the goodness of fit of ODR, the coefficient of determination, R^2 is introduced. R^2 is calculated from the sum of squares of residuals, RSS and the total sum of squares, TSS as,

$$R^2 = 1 - \frac{\mathrm{RSS}}{\mathrm{TSS}}, \tag{25}$$

where RSS and TSS for ODR is calculated as,

$$\mathrm{RSS} = \sum_i d(x_i, y_i, f(\boldsymbol{\beta}))^2 \tag{26}$$

$$\mathrm{TSS} = \sum_i (y_i - \bar{y})^2, \tag{27}$$

where (x_i, y_i) denotes the coordinate of the ith observed data, $d(x_i, y_i, f)^2$ denotes the squared orthogonal distance between (x_i, y_i) and the fitted curve f, and $\bar{y}$ denotes the mean value of y_i.

Flow power plot To fit a curve to the data in figure 3a in the main text, we use the least squares fit for a parametric curve fitting. To simplify notation, here we will use the notation $x = \log_{10}(P_f/Rghw_s)$, $y = \log_{10}(\Phi)$, so that the goal is to fit a curve $(x(t), y(t))$ to the given set of data points (x_i, y_i). The curves are written as polynomials in the parameter t, and we choose to take x as a quadratic in t and y as a cubic in t, so that

$$x = \sum_{n=0}^{2} a_n t^n, \qquad y = \sum_{m=0}^{3} b_m t^m. \tag{28}$$

This parametrization in the variable t has a symmetry of the form $t \mapsto c_0 + c_1 t$, which has corresponding transformations for the coefficients a_n, b_n, that keeps the curve in the x-y plane unchanged. To remove this symmetry, we select two of the coefficients by choosing $t = 0$ to correspond to the extremal value of x, $a_1 = 0$, and the rate of change of y around this point to be unity, $b_1 = 1$.

To fit the curve to the data (x_i, y_i), we recognise that we must not only determine the coefficients a_n, b_n, but also a set of values t_i which are the parameter values for the closest point on the curve to the data point. We do this employing the residuals

$$\varepsilon_i(\boldsymbol{\beta}) = x_i - f_i(\boldsymbol{\beta}) \qquad \text{where} \qquad f_i(\boldsymbol{\beta}) = \sum_{n=0}^{2} a_n t_i^n, \tag{29}$$

and

$$\delta_i(\boldsymbol{\beta}) = y_i - g_i(\boldsymbol{\beta}) \qquad \text{where} \qquad g_i(\boldsymbol{\beta}) = \sum_{m=0}^{3} b_n t_i^m, \tag{30}$$

where bold characters denote column vectors or matrices, and $\boldsymbol{\beta}$ will be defined later. For now we simply state it is the value to be optimised. The sum of squared residuals is then

$$S(\boldsymbol{\beta}) = \sum_i (\varepsilon_i(\boldsymbol{\beta})^2 + \delta_i(\boldsymbol{\beta})^2). \tag{31}$$

and we employ the method of least squares, seeking a local minimum of $S(\boldsymbol{\beta})$. Following the standard deviation for the method of least squares, we can find a local minimum by iterating from one value, $\boldsymbol{\beta}^k$, to the next, $\boldsymbol{\beta}^{k+1}$, using

$$(\boldsymbol{F}^T \boldsymbol{F} + \boldsymbol{G}^T \boldsymbol{G}) \Delta\boldsymbol{\beta} = \boldsymbol{F}^T \boldsymbol{\varepsilon} + \boldsymbol{G}^T \boldsymbol{\delta} \tag{32}$$

where $\boldsymbol{\beta}^{k+1} = \boldsymbol{\beta}^k + \Delta\boldsymbol{\beta}$ and

$$F_{ij} = \frac{\partial f_i}{\partial \beta_j}(\boldsymbol{\beta}^k), \qquad G_{ij} = \frac{\partial g_i}{\partial \beta_j}(\boldsymbol{\beta}^k). \tag{33}$$

The algorithm we use proceeds as follows, where $\|\boldsymbol{\beta}\| = \frac{1}{M}\sum_{m=1}^{M}\beta_m$.

1. Initialise the values of t_i to $t_i = 1$ for $x_i < 1$, $y_i > -2$; to $t_i = 0$ for $x_i \geq 1$; and to $t_i = -1$ otherwise. Add to these t_i a random amount between -0.05 and 0.05 to desingularize what follows.
2. Initialise a_n and b_m to random values between 0 and 1, except for $a_1 = 0$ and $b_1 = 1$.
3. Apply the iteration (32) with $\boldsymbol{\beta} = (a_0, a_2, b_0)^T$ until $\|\Delta\boldsymbol{\beta}\| < 10^{-4}$, which is equivalent to fitting x as a quadratic in y,
4. Apply the iteration (32) with $\boldsymbol{\beta} = (a_0, a_2, b_0, b_2, b_3, t_0, t_1, \ldots)^T$ and $\boldsymbol{\beta}^{k+1} = \boldsymbol{\beta}^k + \frac{1}{10}\Delta\boldsymbol{\beta}$ until $\|\Delta\boldsymbol{\beta}\| < 10^{-4}$, which is the full fitting of the curve
5. For each point, search over values of t to find a t_i that is the global minimizer of $\varepsilon_i^2 + \delta_i^2$, to ensure that all data points are identified with the correct points on the curve
6. Apply step 4 again

At this stage the optimal values of a_n and b_m are known, and can be used to plot the best fit curve. For both cases $a_1 = 0$ and $b_1 = 1$. For the fluvial case

$$\begin{aligned} &a_0 = 0.332, \quad a_1 = 0, \quad a_2 = -1.412, \\ &b_0 = -1.281, \quad b_1 = 1, \quad b_2 = -0.583, \quad b_3 = 0.1621, \\ &-1.282 < t < 1.179. \end{aligned} \tag{34}$$

Supplementary References

1. Pope, S. B. & Pope, S. B. *Turbulent flows* (Cambridge university press, 2000).

2. Michon, X., Goddet, J. & Bonnefille, R. *Etude theorique et experimentale des courants de densite* (Laboratoire national d'hydraulique, 1955).

3. Altinakar, M. S. *Weakly Depositing Turbidity Currents on Small Slopes*. PhD dissertation, EPF Lausanne, CH. (1988).

4. Tesaker, E. *Uniform turbidity current experiments*. PhD dissertation, The Technical University of Norway (1969).

5. Soulsby, R. *Dynamics of marine sands* (T. Telford London, 1997).

6. Garcìa, M. H. Hydraulic jumps in sediment-driven bottom currents. *J. Hydraul. Eng.* **119**, 1094–1117, DOI: 10.1061/(ASCE)0733-9429(1993)119:10(1094) (1993).

7. Packman, A. & Jerolmack, D. The role of physicochemical processes in controlling sediment transport and deposition in turbidity currents. *Mar. Geol.* **204**, 1–9, DOI: 10.1016/S0025-3227(03)00359-1 (2004).

8. Amy, L., Peakall, J. & Talling, P. Density- and viscosity-stratified gravity currents: Insight from laboratory experiments and implications for submarine flow deposits. *Sedimentary Geol.* **179**, 5 – 29, DOI: 10.1016/j.sedgeo.2005.04.009 (2005). Sedimentary Gravity Flows: Recent Advances in Process and Field Analysis.

9. Islam, M. A. & Imran, J. Vertical structure of continuous release saline and turbidity currents. *J. Geophys. Res. Ocean.* **115**, DOI: 10.1029/2009JC005365 (2010).

10. Sequeiros, O. E. *et al.* Bedload transport and bed resistance associated with density and turbidity currents. *Sedimentology* **57**, 1463–1490, DOI: 10.1111/j.1365-3091.2010.01152.x (2010).

11. Sequeiros, O. E. *et al.* Characteristics of velocity and excess density profiles of saline underflows and turbidity currents flowing over a mobile bed. *J. Hydraul. Eng.* **136**, 412–433, DOI: 10.1061/(ASCE)HY.1943-7900.0000200 (2010).

12. Cartigny, M. J., Eggenhuisen, J. T., Hansen, E. W. & Postma, G. Concentration-dependent flow stratification in experimental high-density turbidity currents and their relevance to turbidite facies models. *J. Sedimentary Res.* **83**, 1047–1065, DOI: 10.2110/jsr.2013.71 (2013).

13. Varjavand, P., Ghomeshi, M., Dalir, A. H., Farsadizadeh, D. & Gorgij, A. D. Experimental observation of saline underflows and turbidity currents, flowing over rough beds. *Can. J. Civ. Eng.* **42**, 834–844, DOI: 10.1139/cjce-2014-0537 (2015).

14. Fedele, J. J., Hoyal, D., Barnaal, Z., Tulenko, J. & Awalt, S. Bedforms created by gravity flows. In *Autogenic Dynamics and Self-Organization in Sedimentary Systems*, DOI: 10.2110/sepmsp.106.12 (SEPM Society for Sedimentary Geology, 2016).

15. Breard, E. C. & Lube, G. Inside pyroclastic density currents – uncovering the enigmatic flow structure and transport behaviour in large-scale experiments. *Earth Planet. Sci. Lett.* **458**, 22–36, DOI: 10.1016/j.epsl.2016.10.016 (2017).

16. de Leeuw, J., Eggenhuisen, J. T. & Cartigny, M. J. B. Linking submarine channel–levee facies and architecture to flow structure of turbidity currents: insights from flume tank experiments. *Sedimentology* **65**, 931–951, DOI: https://doi.org/10.1111/sed.12411 (2018).

17. de Leeuw, J. *et al.* Sediment volume and grain-size partitioning between submarine channel-levee systems and lobes: An experimental study. *J. Sedimentary Res.* **88**, 777–794, DOI: 10.2110/jsr.2018.46 (2018).

18. Hermidas, N. *et al.* A classification of clay-rich subaqueous density flow structures. *J. Geophys. Res. Earth Surf.* **123**, 945–966, DOI: 10.1002/2017JF004386 (2018).

19. Sequeiros, O. E., Mosquera, R. & Pedocchi, F. Internal structure of a self-accelerating turbidity current. *J. Geophys. Res. Ocean.* **123**, 6260–6276, DOI: 10.1029/2018JC014061 (2018).

20. Eggenhuisen, J. T., Tilston, M. C., de Leeuw, J., Pohl, F. & Cartigny, M. J. B. Turbulent diffusion modelling of sediment in turbidity currents: An experimental validation of the rouse approach. *The Depositional Rec.* (2019).

21. Farizan, A., Yaghoubi, S., Firoozabadi, B. & Afshin, H. Effect of an obstacle on the depositional behaviour of turbidity currents. *J. Hydraul. Res.* **57**, 75–89, DOI: 10.1080/00221686.2018.1459891 (2019).

22. Kelly, R. W., Dorrell, R. M., Burns, A. D. & McCaffrey, W. D. The structure and entrainment characteristics of partially confined gravity currents. *J. Geophys. Res. Ocean.* **124**, 2110–2125, DOI: 10.1029/2018JC014042 (2019).

23. Koller, D., Manica, R., Borges, A. d. O. & Fedele, J. Experimental bedforms by saline density currents. *Brazilian Journal of Geology* **49**, DOI: 10.1590/2317-4889201920180118 (2019).

24. Brosch, E. & Lube, G. Spatiotemporal sediment transport and deposition processes in experimental dilute pyroclastic density currents. *J. Volcanol. Geotherm. Res.* **401**, 106946, DOI: 10.1016/j.jvolgeores.2020.106946 (2020).

25. Pohl, F., Eggenhuisen, J. T., Kane, I. A. & Clare, M. A. Transport and burial of microplastics in deep-marine sediments by turbidity currents. *Environ. Sci. & Technol.* **54**, 4180–4189, DOI: 10.1021/acs.est.9b07527 (2020).

26. Simmons, S. M. *et al.* Novel acoustic method provides first detailed measurements of sediment concentration structure within submarine turbidity currents. *J. Geophys. Res. Ocean.* **125**, e2019JC015904, DOI: 10.1029/2019JC015904 (2020).

27. Fisher, P., Aumann, C., Chia, K., O'Halloran, N. & Chandra, S. Adequacy of laser diffraction for soil particle size analysis. *PLOS ONE* **12**, 1–20, DOI: 10.1371/journal.pone.0176510 (2017).

28. Goossens, D. Techniques to measure grain-size distributions of loamy sediments: a comparative study of ten instruments for wet analysis. *Sedimentology* **55**, 65–96, DOI: 10.1111/j.1365-3091.2007.00893.x (2008).

29. Eshel, G., Levy, G. J., Mingelgrin, U. & Singer, M. J. Critical evaluation of the use of laser diffraction for particle-size distribution analysis. *Soil Sci. Soc. Am. J.* **68**, 736–743, DOI: 10.2136/sssaj2004.7360 (2004).

30. Katayama, H. Comparison between a lazer diffraction-scattering method and a hydrometer method in the grain size analysis for fine-grained sediments. *J. Sedimentol. Soc. Jpn.* **46**, 23–30, DOI: 10.4096/jssj1995.46.23 (1997).

31. Vitton, S. J. & Sadler, L. Y. Particle-size analysis of soils using laser light scattering and x-ray absorption technology. *Geotech. Test. J.* **20**, 63–73 (1997).

32. Konert, M. & Vandenberghe, J. Comparison of laser grain size analysis with pipette and sieve analysis: a solution for the underestimation of the clay fraction. *Sedimentology* **44**, 523–535, DOI: 10.1046/j.1365-3091.1997.d01-38.x (1997).

33. Lu, N., Ristow, G. H. & Likos, W. J. The accuracy of hydrometer analysis for fine-grained clay particles. *Geotech. Test. J.* **23**, 487–495 (2000).

34. Di Stefano, C., Ferro, V. & Mirabile, S. Comparison between grain-size analyses using laser diffraction and sedimentation methods. *Biosyst. Eng.* **106**, 205–215, DOI: 10.1016/j.biosystemseng.2010.03.013 (2010).

35. Al-Hashemi, H. M. B., Al-Amoudi, O. S. B., Yamani, Z. H., Mustafa, Y. M. & Ahmed, H.-u.-R. The validity of laser diffraction system to reproduce hydrometer results for grain size analysis in geotechnical applications. *PLOS ONE* **16**, 1–18, DOI: 10.1371/journal.pone.0245452 (2021).

36. Lopez, A., Gustavsson, H. & Korkiala-Tanttu, L. Comparison between hydrometer and laser diffraction methods in the determination of clay content in fine-grained soils. *IOP Conf. Series: Earth Environ. Sci.* **710**, 012012, DOI: 10.1088/1755-1315/710/1/012012 (2021).

37. Bah, A. R., Kravchuk, O. & Kirchhof, G. Fitting performance of particle-size distribution models on data derived by conventional and laser diffraction techniques. *Soil Sci. Soc. Am. J.* **73**, 1101–1107, DOI: 10.2136/sssaj2007.0433 (2009).

38. Fritsch, F. N. & Butland, J. A method for constructing local monotone piecewise cubic interpolants. *SIAM J. on Sci. Stat. Comput.* **5**, 300–304, DOI: 10.1137/0905021 (1984).

39. Lavery, A. C., Schmitt, R. W. & Stanton, T. K. High-frequency acoustic scattering from turbulent oceanic microstructure: The importance of density fluctuations. *The J. Acoust. Soc. Am.* **114**, 2685–2697, DOI: 10.1121/1.1614258 (2003).

40. Parker, G., Fukushima, Y. & Pantin, H. M. Self-accelerating turbidity currents. *J. Fluid Mech.* **171**, 145–181, DOI: 10.1017/S0022112086001404 (1986).

41. Velikanov, M. *et al.* Gravitational theory of sediment transport. *J. Sci. Sov. Union, Geophys.* **4** (1954).

42. Bagnold, R. A. *An approach to the sediment transport problem from general physics*, vol. 422 (U.S. Geol. Surv. Prof. Paper, 1966).

43. Bagnold, R. A., Deacon, G. E. R. & Russell, F. S. Auto-suspension of transported sediment; turbidity currents. *Proc. Royal Soc. London. Ser. A. Math. Phys. Sci.* **265**, 315–319, DOI: 10.1098/rspa.1962.0012 (1962).

44. Dellino, P., Dioguardi, F., Doronzo, D. M. & Mele, D. The entrainment rate of non-boussinesq hazardous geophysical gas-particle flows: An experimental model with application to pyroclastic density currents. *Geophys. Res. Lett.* **46**, 12851–12861, DOI: 10.1029/2019GL084776 (2019).

45. Nordin, C. F. & Dempster, G. R. *Vertical distribution of velocity and suspended sediment, Middle Rio Grande, New Mexico*. 462-B (US Government Printing Office, 1963).

46. Wan, Z. H. & Wang, Z. Y. Hyperconcentrated flow, IAHR monograph series. *Balkema: Rotterdam* (1994).

47. Guy, H., Simons, D. & Richardson, E. Summary of alluvial channel data from flume experiments, 1956-61: Us geol. *Surv. Prof. Pap.* **462**, 11–196 (1966).

48. Ashida, K. & Okabe, T. On the calculation method of the concentration of suspended sediment under non-equilibrium condition. *Proceedibgs if Jpn. conference on hydraulics* **26**, 153–158, DOI: 10.2208/prohe1975.26.153 (1982).

49. Cellino, M. & Graf, W. H. Sediment-laden flow in open-channels under noncapacity and capacity conditions. *J. Hydraul. Eng.* **125**, 455–462, DOI: 10.1061/(ASCE)0733-9429(1999)125:5(455) (1999).

50. Graf, W. & Cellino, M. Suspension flows in open channels; experimental study. *J. Hydraul. Res.* **40**, 435–447, DOI: 10.1080/00221680209499886 (2002).

51. Einstein, H. & Chien, N. Effects of heavy sediment concentration near the bed on velocity and sediment distribution. mrd sediment series no. 8. *Univ California, Berkeley, US Army Corps Eng. Mo. Div* (1955).

52. Coleman, N. L. Effects of suspended sediment on the open-channel velocity distribution. *Water Resour. Res.* **22**, 1377–1384, DOI: https://doi.org/10.1029/WR022i010p01377 (1986).

53. Lyn, D. A. A similarity approach to turbulent sediment-laden flows in open channels. *J. Fluid Mech.* **193**, 1–26, DOI: 10.1017/S0022112088002034 (1988).

54. Vanoni, V. A. Transportation of suspended sediment by water. *Transactions Am. Soc. Civ. Eng.* **111**, 67–102, DOI: 10.1061/TACEAT.0005975 (1946).

55. Vanoni, V. A. & Nomicos, G. N. Resistance properties of sediment-laden streams. *Transactions Am. Soc. Civ. Eng.* **125**, 1140–1167, DOI: 10.1061/TACEAT.0007902 (1960).

56. Brooks, N. H. *Laboratory studies of the mechanics of streams flowing over a movable bed of fine sand*. Ph.D. thesis, California Institute of Technology (1954). DOI: 10.7907/JB2P-1H91.

57. van Maren, D. Grain size and sediment concentration effects on channel patterns of silt-laden rivers. *Sedimentary Geol.* **202**, 297–316, DOI: 10.1016/j.sedgeo.2007.04.001 (2007).

58. Moodie, A. J. *et al.* Suspended sediment-induced stratification inferred from concentration and velocity profile measurements in the lower yellow river, china. *Water Resour. Res.* **58**, e2020WR027192, DOI: https://doi.org/10.1029/2020WR027192 (2022).

59. Dorrell, R. M., Amy, L. A., Peakall, J. & McCaffrey, W. D. Particle size distribution controls the threshold between net sediment erosion and deposition in suspended load dominated flows. *Geophys. Res. Lett.* **45**, 1443–1452, DOI: 10.1002/2017GL076489 (2018).

60. Boggs, P. T. & Rogers, J. E. Orthogonal distance regression. *Contemp. Math.* **112**, 183–194 (1990).
